# Towards standardization in pig microbiome research based on a comprehensive twenty-year review

**DOI:** 10.1186/s42523-026-00541-0

**Published:** 2026-03-09

**Authors:** Samuel Onyilokwu Enokela, Timur Yergaliyev, Krzysztof Flisikowski, Stéphanie Céline Hornburg, Henry Reyer, Jens Tetens, Klaus Wimmers, Jürgen Zentek, Amélia Camarinha-Silva

**Affiliations:** 1 Institute of Animal Science, University of Hohenheim, Emil-Wolff-Str. 10, Stuttgart, Germany; 2 Infection Pathogenesis, Technical University of Munich, Liesel-Beckmann-Straße 1, Freising, Germany; 3 Institute for Animal Nutrition and Physiology, Christian-Albrechts University, Hermann-Rodewald-Straße 9, Kiel, Germany; 4 Research Institute for Farm Animal Biology (FBN), Wilhelm-Stahl-Allee 2, Dummerstorf, Germany; 5 Department of Functional Breeding, Georg-August University, Burckhardtweg 2, Göttingen, Germany; 6 Institute of Animal Nutrition, Freie Universität-Berlin, Königin-Luise-Str. 49, Berlin, Germany; 7 Hohenheim Center for Livestock Microbiome Research, University of HohenheimLeonore-Blosser-Reisen Weg 3, 70599, Stuttgart, Germany

**Keywords:** Metadata, Pig, Standardization, Microbiome

## Abstract

The pig microbiome plays a crucial role in animal health, productivity, and meat quality. This comprehensive review analyzed 438 publications on pig microbiome studies from 2003 to 2023, focusing on experimental methodologies, bioinformatic tools, sequencing approaches, and metadata reporting practices. It critically analyzes the variability introduced by different DNA extraction methods, sequencing platforms, and bioinformatics pipelines, emphasizing their impacts on reproducibility and data comparability. We identify critical gaps in metadata reporting, particularly in DNA extraction protocols and bioinformatic workflows. To address these issues, we propose a standardized metadata template for pig microbiome studies, integrating Genomic Standards Consortium guidelines with project-specific criteria. This review provides a framework for developing standardized protocols in pig microbiome research, aiming to improve reproducibility and facilitate cross-study comparisons.

**Supplementary Information:**

The online version contains supplementary material available at 10.1186/s42523-026-00541-0.

## Introduction

The gut microbiome of pigs maintains metabolic homeostasis, supports immune function, and influences growth and feed efficiency [1]. Understanding the composition and function of this complex microbial community is increasingly recognized as a key factor in improving swine health and production outcomes. In livestock, the intestinal microbiome significantly influences productivity and meat quality traits [2]. The microbial genetic diversity within the gastrointestinal tract (GIT) facilitates diverse biological processes complementary to host functions, such as nutrient metabolism and immune modulation. Moreover, the microbiota offers insights into the physiological state of animals and the environmental factors affecting their performance, thus serving as a potential biomarker [3].

Pigs, a critical source of meat for human consumption, whose production reached 362.6 metric tons in 2022 [4], are prominently featured in microbiome studies due to their dual importance in agriculture and biomedical research [5]. Understanding the porcine gut microbiome composition and functionality across intestinal sections is important for improving animal health management and enhancing production and performance practices [6]. Recent research has shown the significant impact of host genetics on gut microbial composition, directly influencing microbial abundance in pigs [6]. Camarinha-Silva et al. (2017) [7] demonstrated that microbial abundances can be used to predict complex traits in pigs with a higher accuracy than genomic predictions. Supporting this, Aliakbari et al. (2022) [8] identified significant correlations between fecal microbial composition and performance traits in pigs such as the average daily gain, backfat thickness, feed conversion ratio, and residual feed intake, showing prediction accuracies ranging from 0.60 to 0.78. Research by Lu et al. (2024) [9] also emphasized the combined contribution of genetic and microbial factors to phenotypic variations in pig growth and feed efficiency. Furthermore, the pig gut microbiome significantly influences immunity traits, including lymphocyte phagocytic capacity, demonstrating moderate heritability and microbiability, showing the combined influence of host genetics and gut microbiota on the immune system [10]. Environmental, host, and management factors such as housing, stress, diet, medicines, sex, and age are also known to influence the constitution of the gut microbiome [11, 12]. For example, studies have shown that different housing systems, feed types, and management practices can lead to distinct microbial profiles, which in turn affect animal performance and health outcomes [13–15]. Moreover, the pig gut microbiome is not static; it undergoes dynamic changes throughout development, particularly during the transition from nursing to weaning, when the microbial community stabilizes and matures [16].

While the field has made significant improvements, methodological inconsistencies remain a major challenge. Microbiome research is critically dependent on the accuracy of the wet-lab protocols, bioinformatic tools, and pipelines used to analyze sequencing datasets. For instance, differences in DNA extraction protocols can affect the recovery of certain microbial taxa, particularly those with robust cell walls, and the choice of sequencing region or platform can influence the resolution and accuracy of taxonomic assignments [17]. The need for standardization is shown by studies demonstrating how DNA extraction methods influence microbial diversity and composition. A study by Burbach et al. (2016) [18] found that mechanical lysis was critical for recovering DNA from Gram-positive bacteria in pig feces. Similarly, Galla et al. (2024) [19] highlighted that inconsistencies in DNA extraction protocols led to significant variation in alpha and beta diversity metrics across studies. Reagent contamination can introduce exogenous DNA to low biomass samples and introduce biases or false positives in microbial composition analyses [20]. Additionally, laboratory-specific workflows and the degree of automation in DNA extraction stages contribute to variability, making cross-study comparisons challenging ^20^. Automation of DNA extraction stages is particularly important for large-scale studies, as it enhances reproducibility and throughput while reducing human error [20]. For example, automated systems like the QIAcube system (Qiagen) and the NucliSens miniMAG extraction instrument have been successfully employed in microbiome studies [21, 22] and could be adapted for pig microbiome research. While numerous standardized protocols exist for extracting microbial DNA from human samples, particularly feces [20], similar comprehensive evaluations of DNA extraction protocols for pig microbiomes do not exist. The Human Microbiome Project (HMP) [23] and MetaHIT [24] have standardized DNA extraction methods for human studies, emphasizing the need to reduce variability between protocols. These initiatives standardized methods to minimize biases introduced by different lysis techniques, reagent contamination, and other procedural inconsistencies.

Differences in bioinformatic pipelines and tools can also significantly influence microbial profile composition, leading to biases that affect cross-study comparisons and downstream analyses [25, 26]. Studies have shown that variations in software, algorithms, and parameter settings directly affect microbial community metrics and taxonomic annotations [27–30]. The 16S rRNA gene (16S RGA), approximately 1500 bp in length, is used for microbial identification and profiling due to its highly conserved regions interspersed with nine variable regions (V1–V9), which provide taxonomic specificity (Chakravorty et al., 2007). However, the choice of variable regions targeted for amplification can significantly influence sequencing outcomes and taxonomic annotations. Studies have shown that the targeted region impacts microbial diversity assessments, as some regions may provide better resolution for certain taxa while failing to identify others [26, 31, 32].

Recognizing these challenges, the microbiome research community has developed reporting guidelines and metadata standards, such as the Minimum Information about any (x) Sequence (MIxS) checklist and the STORMS guidelines, to promote transparency, reproducibility, and data sharing in microbiome studies [33]. However, adoption of these standards in pig microbiome research remains uneven, and critical gaps persist in the documentation of experimental and analytical details. Thus, this study aims to evaluate relevant literature on the pig microbiome to identify trends on metadata reporting and to determine the most used DNA extraction protocols, 16S RGA regions, bioinformatic tools, taxonomic and functional tools and databases.

This review systematically examined 438 publications on pig microbiome studies published between 2003 and 2023. We assess current methodologies, sequencing approaches, bioinformatic tools, and metadata reporting practices, with a particular focus on identifying sources of variability that impact reproducibility and data integration. Based on our findings, we propose a comprehensive metadata template tailored to pig microbiome research, aiming to facilitate standardization, improve cross-study comparability, and support future advances in the field.

## Data collection and eligibility criteria for the reviewed literature

**Fig. 1:**
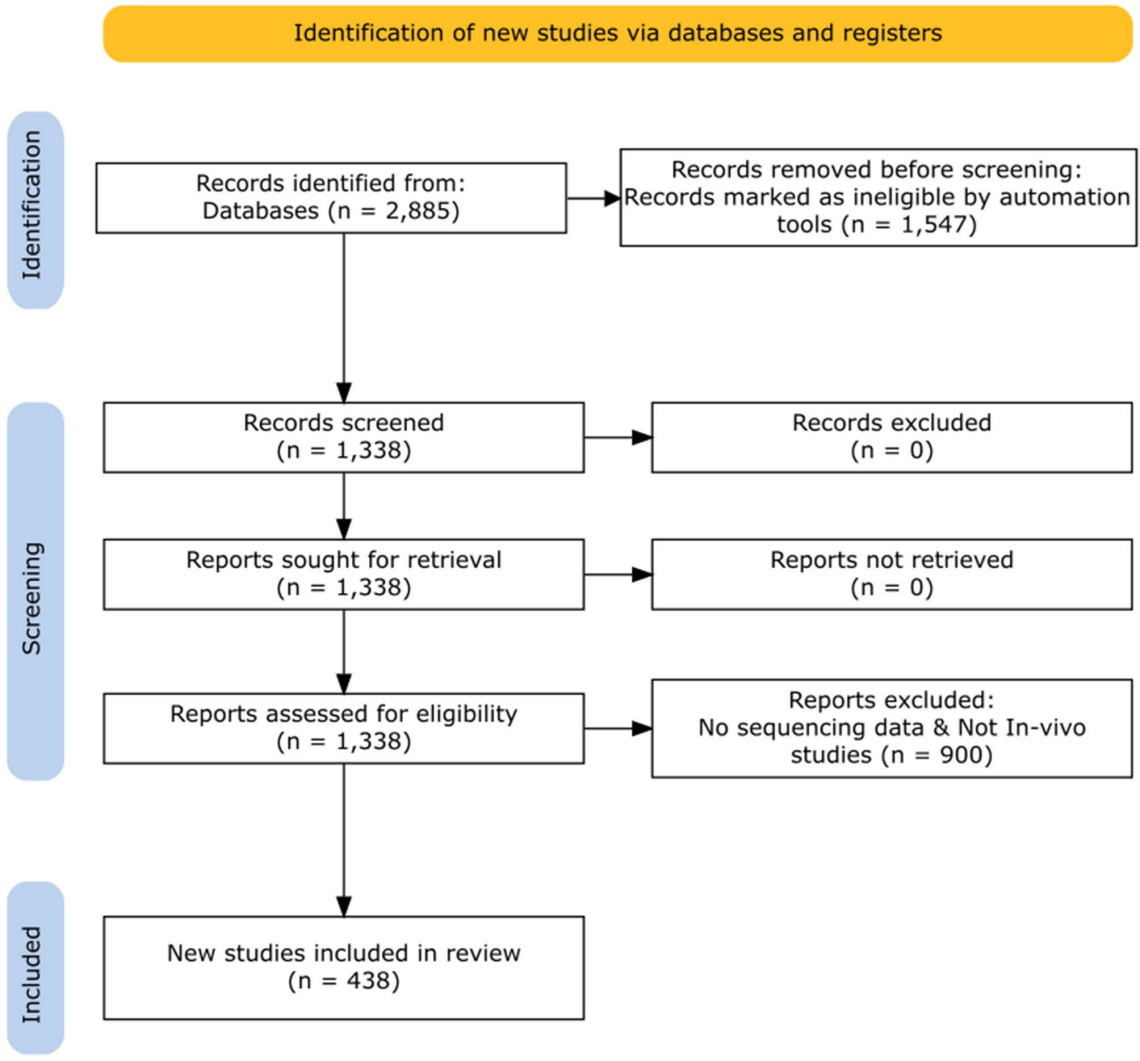
PRISMA flowchart of database search results and exclusion criteria

Following Preferred Reporting Items for Systematic reviews and Meta-Analyses (PRISMA) guidelines [34, 35], we compiled the dataset for this review by combining the results of several keyword-based searches conducted in the PubMed database. This database was selected for its focus on life science and biomedical literature and its accessibility as an open resource. Databases like Web of Science or Scopus were not included due to the close alignment between PubMed’s scope and the objectives of this review. The literature searches targeted publications containing the terms “pig microbiome” and “pig microbiota,” published between January 1st, 2003, and April 30th, 2023, and available as full-text articles, and this initial query retrieved 2,885 publications. To focus on studies with sequencing data, an automated filter was applied to identify publications that mentioned sequencing information within their text. This step reduced the pool to 1,338 studies, while 1,547 were excluded for lacking relevant sequencing data. Further manual screening was conducted on these 1,338 studies using the following eligibility criteria: (1) the study evaluated the gut microbiota of pigs under any experimental or environmental condition, (2) the study used 16S RGAS or shotgun metagenomics methodologies, and (3) review articles and in vitro studies were excluded (Fig. 1).

An additional requirement was that sequencing data from each eligible study had to be publicly available and accessible as of April 31, 2023, when the search was conducted. This ensured that all studies included in the review could be independently verified and reanalyzed, supporting reproducibility. After applying these criteria, a final set of 438 publications (Supplementary File 1) was selected for detailed review.

**Fig. 2:**
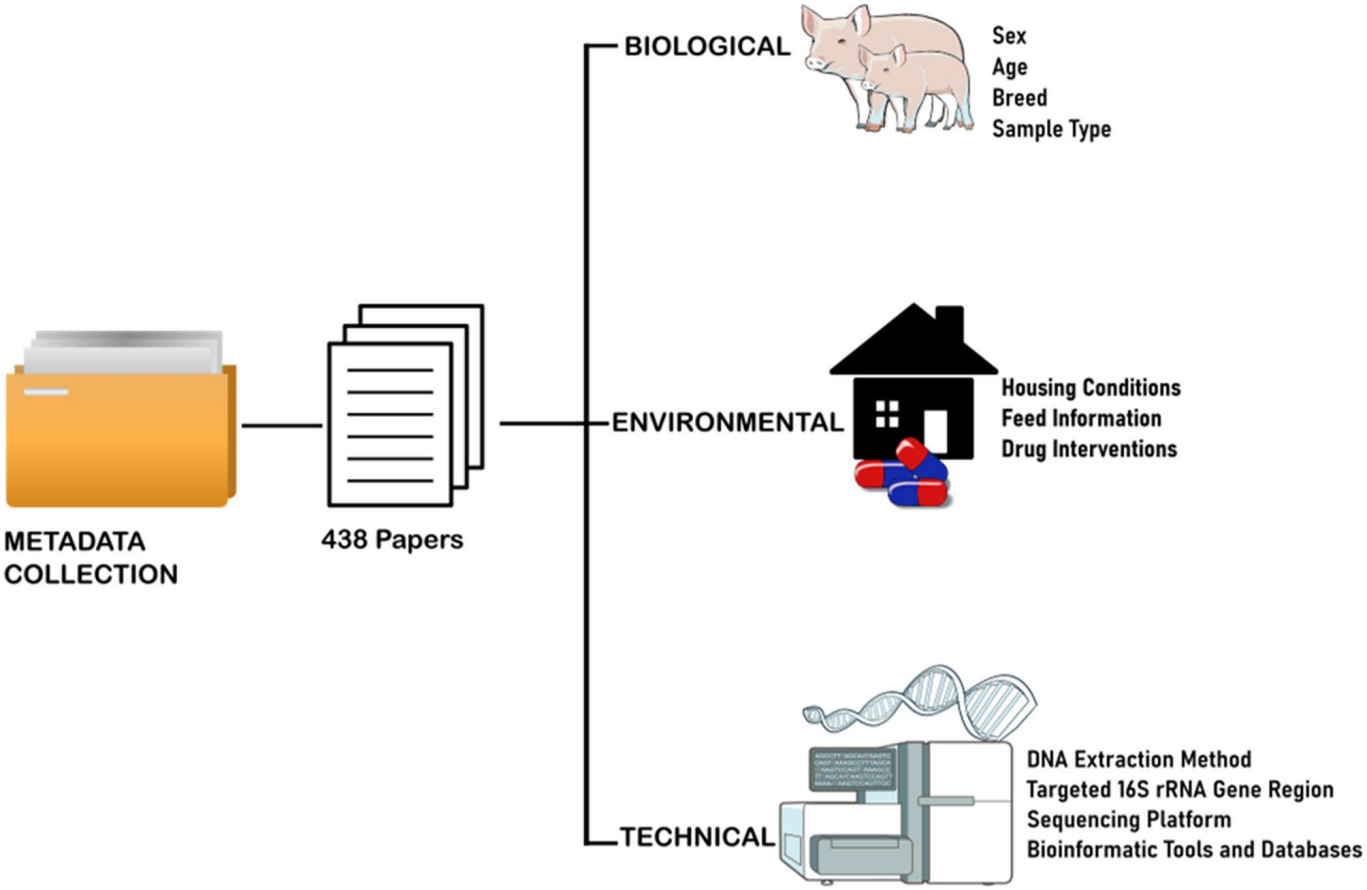
Overview of metadata categories reviewed from selected publications

Because metadata completeness is critical to reproducibility in microbiome research, each selected publication was also evaluated for metadata reporting. The metadata extraction process focused on experimental design, technical protocols, and biological context, adhering to the Minimum Information about any (x) Sequence (MIxS) checklists and related standards [36, 37]. This approach ensured compliance with FAIR data principles (Findable, Accessible, Interoperable, and Reusable) [38]. The rationale is that missing or incomplete metadata limits the scope and accuracy of downstream analyses or retrospective meta-analyses [39]. Figure 2 provides a schematic overview of the metadata categories reviewed from the selected publications.

In summary, this two-step filtering and metadata review process ensured that the studies included in this review were not only methodologically relevant but also met high standards for data accessibility and reporting, enabling a comprehensive and reproducible assessment of current practices in pig microbiome research.

## Current practices and variability in pig microbiome studies

### Study design

A robust study design is indispensable for minimizing errors and clarifying ambiguous trends often encountered in microbiome studies, particularly given the numerous environmental and biological factors that influence the pig microbiome [40]. Consequently, the choice of design directly affects the ability to capture microbial variation accurately and ensure reproducibility. In the literature reviewed, several main types of study design were observed - cohort or longitudinal studies, cross-sectional studies, case-control studies, crossover studies, and randomized controlled trials; each with distinct advantages and limitations.

From a statistical perspective, cohort or longitudinal studies are preferred for analyzing samples under different controlled conditions, as they allow for observing temporal changes in the microbiome within the same population or individuals under controlled conditions. These designs make it possible to assess dynamic interactions between the microbiome and environmental or host factors. However, they have limitations, because cohort studies are susceptible to cofounding variables, can be challenging to apply to rare outcomes, and are often time-consuming and resource-intensive due to the need for long-term follow-up and the risk of participant loss, which can introduce bias [41]. In contrast, cross-sectional studies involve comparative analyses of two or more groups at a single point in time and are less demanding in terms of duration and resources. Although cross-sectional studies provide an image of microbiome differences, they do not capture temporal dynamics or allow for causal inference [42].

Case-control studies are observational in nature and allow for comparability. These studies are conducted to examine risks for rare diseases, isolate risk factors for diseases, generate postulations of risk factors associated with the development of a disease, and also be used to assess harm in situations where experimental studies may be unethical [43]. The major disadvantages of this study type include but are not limited to the reliance on recollection from participants which can lead to recollection bias, selection bias i.e. difficulty in finding a suitable and comparable control group, the inability of the study type to study diseases with low exposure rates, temporal ambiguity when it comes to assessing if exposure occurred before disease developed, and lastly, case-control studies can only be used to establish associations, not causations [43–46].

Crossover design studies, though less commonly utilized in pig microbiome research, provide a unique advantage by exposing the same subjects to different treatments sequentially, thereby reducing between-subject variability ^39^. Randomized controlled trials (RCTs) are considered the gold standard for determining causal relationships, as randomization minimizes confounding from both known and unknown sources. Nevertheless, RCTs are highly controlled, often resource-intensive, and may have limited generalizability due to strict inclusion and exclusion criteria and the artificiality of experimental conditions [47].

The distribution of study designs in the 438 reviewed publications reflects a clear preference for cohort-based approaches, with 256 cohort studies, 89 cross-sectional studies, 58 randomized controlled trials, 22 crossover studies, and 13 case-control studies included. This pattern suggests that researchers prioritize designs that can capture temporal changes and control for confounding factors, which is particularly important given the dynamic nature of the pig gut microbiome.

Sample size and statistical power are often constrained by budget and animal availability, limiting the ability to detect subtle microbiome effects. Temporal and spatial variability in the pig microbiome, driven by factors such as age, diet, environment, and management practices, requires careful matching and control of these variables across study groups. Additionally, inconsistent reporting of study design and metadata limits reproducibility and hampers the ability to compare results across studies [40]. Best practices in microbiome research emphasize the inclusion of both biological and technical controls to ensure data quality and reliability. Technical controls such as sampling blanks, extraction blanks, and PCR blanks are crucial for identifying contamination or technical variability, while positive controls like mock communities help standardize analyses and validate results [40].

### Sample collection and preservation

**Fig. 3:**
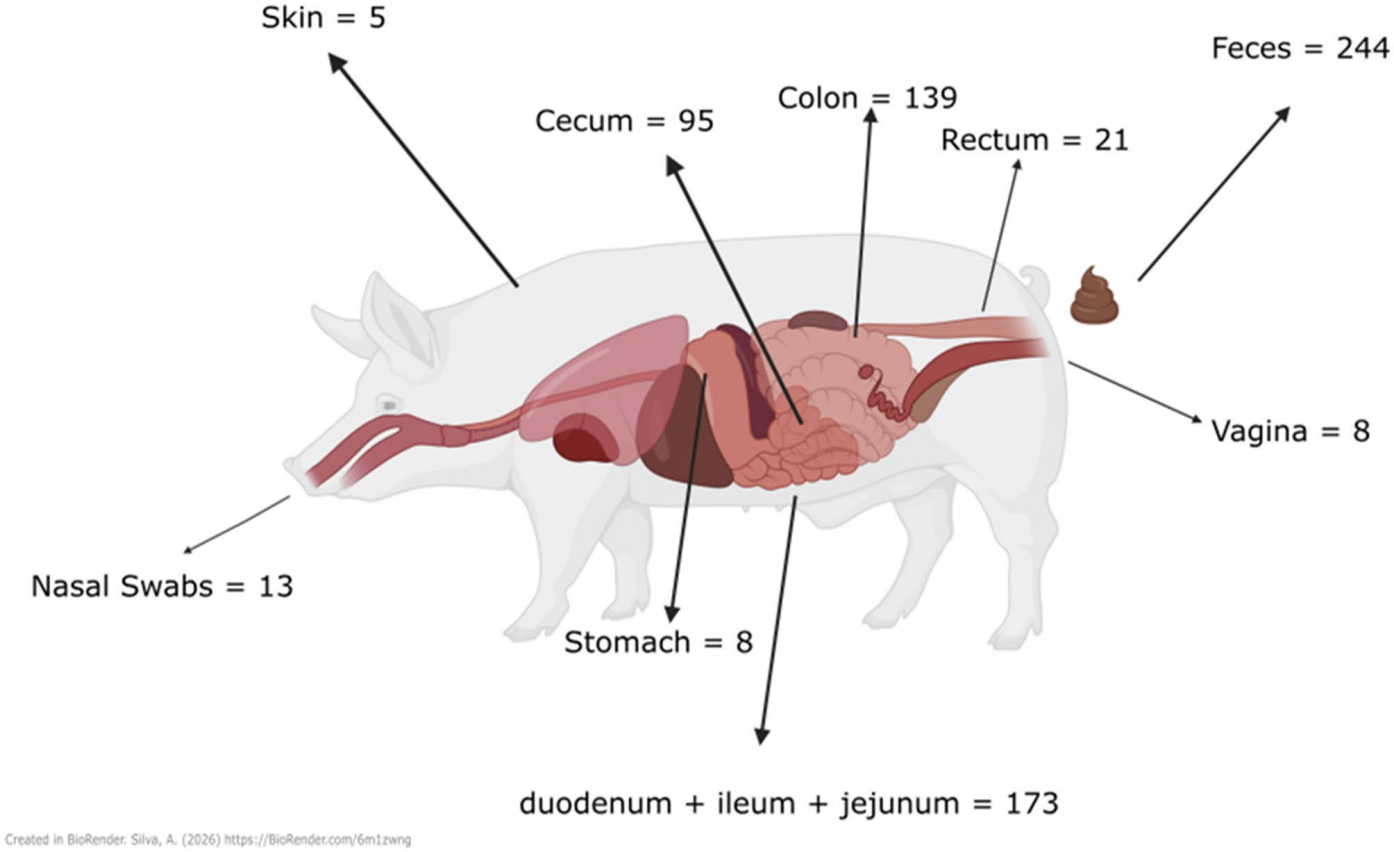
Most common sample types in reviewed publications

Sample collection and preservation are important steps in microbiome research, as they directly impact the integrity and representativeness of microbial communities [48]. Variations in collection techniques can introduce systematic bias depending on the sampling environment [47]. For instance, the physical consistency of fecal samples, such as diarrheic versus formed stools, has been shown to significantly impact microbiota richness and composition [49], emphasizing the need for careful documentation and standardization of sample consistency in gut microbiota studies. From the studies reviewed, fecal samples were by far the most used sample type, appearing in 244 publications, followed by colonic samples (139 studies), with other gastrointestinal tract sections such as the ileum, cecum, jejunum, and duodenum used in a total of 173 studies (Fig. 3). Despite the dominance of fecal sampling, it is important to recognize that fecal microbiota may not fully represent the microbial communities present throughout the gastrointestinal tract, as significant differences can arise due to physiological and environmental factors influencing excretion [50]. Consistency in sample collection methods within a given study is, therefore, a target to minimize variability and enhancing reproducibility.

Preservation methods further influence the microbial profiles recovered. Different stabilization buffers can have varying effects on DNA yield and community structure. For example, samples stored in phosphate-buffered saline (PBS) yielded different microbial compositions compared to RNA-later [51], with some studies reporting decreased DNA purity and diversity in RNA-later-preserved samples [52]. However, other research found that RNA-later can provide higher DNA yields compared to liquid nitrogen or refrigeration at -30 °C, and may not significantly alter alpha or beta diversity in certain contexts [53, 54]. Ethanol is another widely used preservative due to its low cost and accessibility, but results regarding its effectiveness are inconsistent; while 70% ethanol is commonly used for morphological preservation, its ability to stabilize bacterial communities remains inconclusive, whereas 95% ethanol appears to better preserve DNA by rapidly permeating cell membranes and inhibiting DNase activity [48, 55–57].

Several studies have shown that Fast Technology for Analysis of nucleic acids (FTA) cards or Fecal Occult Blood Tests (FOBT) cards are effective DNA stabilizers [48]. These cards possess a remarkable stability over extended periods of time at room temperature [56], and are effective at recovering a wider diversity of bacterial taxa compared to other storage media and buffers [56, 57], particularly spore-forming Bacillota (previously Firmicutes), possibly due to enhanced chemical lysis [56]. Omnigene.Gut buffer has also been shown to preserve DNA integrity for up to 14 days at room temperature without significant changes in microbiota composition compared to samples stored at -80 °C [56, 58, 59].

Created in BioRender. Silva, A. (2026) https://BioRender.com/6m1zwng.

After sample collection, there is a need for proper preservation and storage to maintain microbial integrity for subsequent DNA extraction and sequencing. Adequate DNA quantity and quality are necessary to represent the microbial community accurately and support sequencing library preparation [60]. It has been demonstrated that fecal microbiota composition can change significantly within 48 h at ambient temperature^63^, while storage at -20 °C or -80 °C, if not subjected to more than four freeze-thaw cycles, generally preserves community structure over time [61–63], although some studies have noted an increased *Firmicutes* to *Bacteroidetes* ratio in frozen samples, likely due to greater DNA stability in Gram-positive bacteria [64, 65]. Among the reviewed studies, 349 studies documented the storage temperatures prior to analysis. Of these, 288 studies stored samples at -80 ^o^ C, and 53 studies stored samples at -20 °C, and very few relied on other temperatures. These findings indicate that most studies adopt storage practices aligned with recommendations from scientific literature to preserve bacterial communities effectively.

In conclusion, while fecal samples remain the most common sample type in pig microbiome research, their limitations as proxies for the entire gut microbiota show the need to align sample type with study objectives. Proper storage conditions are critical for preserving microbial integrity, with − 80 °C being the most recommended temperature. Accurate reporting of these parameters as metadata is necessary for reproducibility and comparability. Standardizing collection and preservation practices can help mitigate batch effects, and support meta-analyses.

### DNA extraction protocols

The accuracy and reproducibility of pig gut microbiota studies are strongly influenced by the DNA extraction protocols employed. Variations in these protocols among research organizations contribute to technical discrepancies that complicate direct comparisons between different studies [22]. According to the International Human Microbiome Standards (IHMS), DNA extraction, especially from fecal samples, is recognized as a major source of variability in microbiome research [66], with even minor modifications, such as inclusion of mechanical lysis steps, substantially impacting the observed microbial composition. This is particularly important for Gram-positive bacteria, which have thicker cell walls requiring higher mechanical force for disruption [66, 67]. Concurrently, having longer incubation times improved the efficiency of cell lysis [68].

Comparative studies have demonstrated that different commercial DNA extraction kits yield varying DNA quantities and qualities, as well as distinct microbial community profiles. For example, protocols incorporating bead-beating or mechanical lysis steps tend to improve the recovery of DNA from Gram-positive taxa, such as members of the Bacillota and Actinomycetota, compared to enzyme-based or less rigorous methods [69]. Lu et al. (2015) [70] compared commercial kits (FastDNA Spin Kit and PowerSoil Kit) with a conventional hexadecyltrimethylammonium bromide (CTAB) extraction method, finding that commercial kits generally produced moderate to high DNA yields with high purity (A260/A280 ratios close to 1.8), though some samples showed signs of DNA degradation. In contrast, the conventional CTAB method resulted in greater variability and, in some cases, lower DNA quality. Further, Burbach et al. (2016) [18] evaluated the FastDNA Spin Kit and its variant, the FastDNA Spin Kit for Soil, alongside other commercial kits in pig microbiome studies. Their results indicated that some commercial protocols yielded A260/A280 ratios below 1, suggesting contamination with reagents or proteins. Even among kits from the same manufacturer, performance can vary, for example, the PowerFecal DNA Isolation Kit provided more accurate and consistent DNA yields than the QIAamp DNA Stool Kit, both from Qiagen [2]. However, it has been reported that higher DNA yield does not necessarily correspond to greater community richness or more accurate representation of microbial composition ^74^. The presence of PCR inhibitors, such as humic substances, bile salts, polysaccharides, and hemoglobin, which may be co-extracted with DNA from various sample types, can reduce PCR sensitivity or cause false negatives [18]. To address this, researchers may need to modify extraction steps, switch commercial kits, or incorporate additional procedures aimed explicitly at removing inhibitors [71]. Schrader et al. [71] have outlined various strategies for inhibitor removal tailored to different sample types in pig microbiota studies. Another source of concern is environmental contamination. DNA contaminants have been detected in water from areas where samples were collected [72]. Bacteria identified from these contaminants often belong to soil- or water-dwelling bacteria involved in nitrogen fixation [72].

DNA extraction methodologies can be categorized into three main approaches. The first is the traditional manual method, which relies on chemicals such as ethanol and phenol-chloroform to precipitate nucleic acids [73]. While this method is cost-effective and can yield high-quality DNA, it is unsuitable for large sample sets due to its labor-intensive nature and multiple transfer steps, which increase the risk of contamination and user exposure to hazardous chemicals like phenol. The second category comprises commercially available extraction kits. These kits have a long track record of success and utilize various strategies to isolate nucleic acids, including cell lysis with protein precipitation, salt precipitation, silica columns, and magnetic beads [74]. The performance of these kits can vary substantially depending on the manufacturer, with differences in processing time, cost, input sample type, and resulting nucleic acid yield. While commercial kits are generally less laborious than manual methods and provide more standardized protocols, their manual operation still limits their suitability for high-throughput applications. Additionally, the use of proprietary reagents and consumables can increase costs [74]. The third category involves automated laboratory instruments designed for DNA extraction. These systems are engineered to increase sample processing capacity, throughput, and reproducibility, while also reducing hands-on time and the potential for human error [74]. Automated platforms typically operate via two main mechanisms: binding nucleic acids to a silica membrane or using silica-coated magnetic beads, often based on proprietary chemical processes such as Boom technology [74, 75]. The growing demand for next-generation sequencing in microbiome research has driven the development of several robotic platforms that offer medium- to high-throughput extraction capabilities, combined with generic DNA extraction chemistries to ensure sensitivity, purity, and efficiency [76]. Comparative studies have shown that automated extraction methods generally offer higher throughput but may yield lower DNA quantities and sensitivity compared to manual methods; however, manual extractions are more labor-intensive and require variable processing times depending on the kit and protocol used [68, 77]. Importantly, both manual and automated approaches, when properly optimized, can yield DNA free of PCR inhibitors. As highlighted by Dauphin et al. (2009) [68] the choice between commercial automated and manual DNA extraction methods should be guided by a balance between cost, processing time, throughput, and DNA yield, tailored to the specific needs and scale of the research project.

Accurately determining the concentration of nucleic acids is a requirement in modern molecular laboratories and is closely linked to the efficiency of DNA extraction protocols. For downstream applications such as PCR, library preparation, or sequencing to function optimally, it is required that DNA concentrations fall within a specific range [74]. Inaccurate quantification can introduce variability, undermining the reliability and reproducibility of results [78]. DNA purity is typically assessed using absorbance ratios. The A260/A280 ratio, measured on a scale with a maximum value of 2.0, is widely accepted as an indicator of protein contamination, with values of ≥ 1.8 generally considered to reflect high-purity DNA. However, the A260/A230 ratio, though often overlooked, also provides important information about DNA quality. Absorbance at 230 nm (A230) is sensitive to a range of chemical contaminants, including chaotropic salts and residual extraction reagents, which can inhibit PCR, especially in multiplex reactions, by absorbing light in the 230 nm range and below. Therefore, maintaining low absorbance at 230 nm is important for confirming DNA sample purity and ensuring suitability for downstream applications [74]. It is important to recognize that both biological and chemical contaminants can distort DNA quantification and quality. For instance, chaotropic salts, RNA (when measuring DNA), and proteins can falsely elevate DNA concentration readings. Buffer components such as Tris, Ethylenediaminetetraacetic acid (EDTA), and guanidine isothiocyanate also absorb strongly at 230 nm and can bleed into the 260 nm range, artificially increasing both the A260/A280 and A260/A230 ratios and giving a misleading impression of sample purity [74, 78].

Given these challenges, our review sought to systematically compile detailed information on the DNA extraction methods and kits used in pig microbiome studies, with particular attention to the lysis protocols employed. This approach enables a better understanding of how extraction and quantification practices impact data quality and reliability across studies.

Our analysis revealed that the majority of pig microbiome studies relied on commercial DNA extraction kits (373 studies), which are designed to provide standardized and reproducible protocols, often with built-in variations in lysis techniques to accommodate different sample types. While the use of these kits facilitates consistency across laboratories and simplifies the extraction process, a possible limitation is the lack of detailed reporting on protocol modifications, particularly regarding the lysis step. Many studies do not specify whether they adhered strictly to the manufacturer’s instructions or introduced changes, such as altering bead-beating duration, using different bead types, or modifying buffer compositions, that can significantly influence DNA yield and the representation of specific microbial taxa. Table 1 itemizes the most frequently used commercial DNA extraction kits and methodologies in the reviewed publications.

**Table 1:** Overview of the most used DNA extraction kits and methods in pig microbiome studies

Kit/Method	Description	Number of Studies
QIAamp DNA Stool Mini Kit and Fast DNA Stool Mini Kit	The QIAamp DNA Stool Mini Kit is the most frequently used kit; recognized by IHMS for optimal performance in microbiome analysis with protocol modifications. No longer manufactured, replaced by QIAamp Fast DNA Stool Mini Kit.	81 studies (QIAamp DNA Stool Mini Kit); 31 studies (QIAamp Fast DNA Stool Mini Kit)
Phenol-Chloroform Method	Second most common protocol. Cost-effective but involves hazardous chemicals, labor-intensive, and time-consuming procedures.	47 studies
E.Z.N.A Stool and Soil DNA Kits	High-throughput kit suitable for large-scale DNA extractions; limitations include a lengthy protocol and higher cost compared to Qiagen kits.	39 studies
MO BIO Laboratories Kits	PowerSoil and PowerFecal DNA Isolation Kits, widely used in earlier studies. Replaced by QIAamp PowerFecal Pro DNA Kit with improved inhibitor elimination and enhanced bacterial lysis features.	24 studies (PowerSoil DNA Isolation Kit); 24 studies (PowerFecal DNA Isolation Kit)
Unavailable	Studies which provided no information about the protocol or commercial kit used in DNA extraction	33 studies

### Targeted 16 S rRNA gene regions and shotgun metagenomics

Our review found that 88% of the reviewed pig microbiome studies employed 16S rRNA sequencing (16S RGAS) as their primary approach for profiling microbial communities, while only 12% used shotgun metagenomics. 16S RGAS is widely favored for its cost-effectiveness and high-throughput capabilities, making it accessible for studies with moderate to large sample sizes. However, this approach is not without limitations. The taxonomic resolution of 16S RGAS is inherently restricted by the conserved nature of the gene and the limited variability within the amplified regions [79]. Furthermore, PCR amplification can introduce artifacts that bias the quantification of microbial taxa [80]. Additionally, except for full-length sequencing, no single variable region provides a comprehensive depiction of bacterial diversity at the species level [81].

**Fig. 4:**
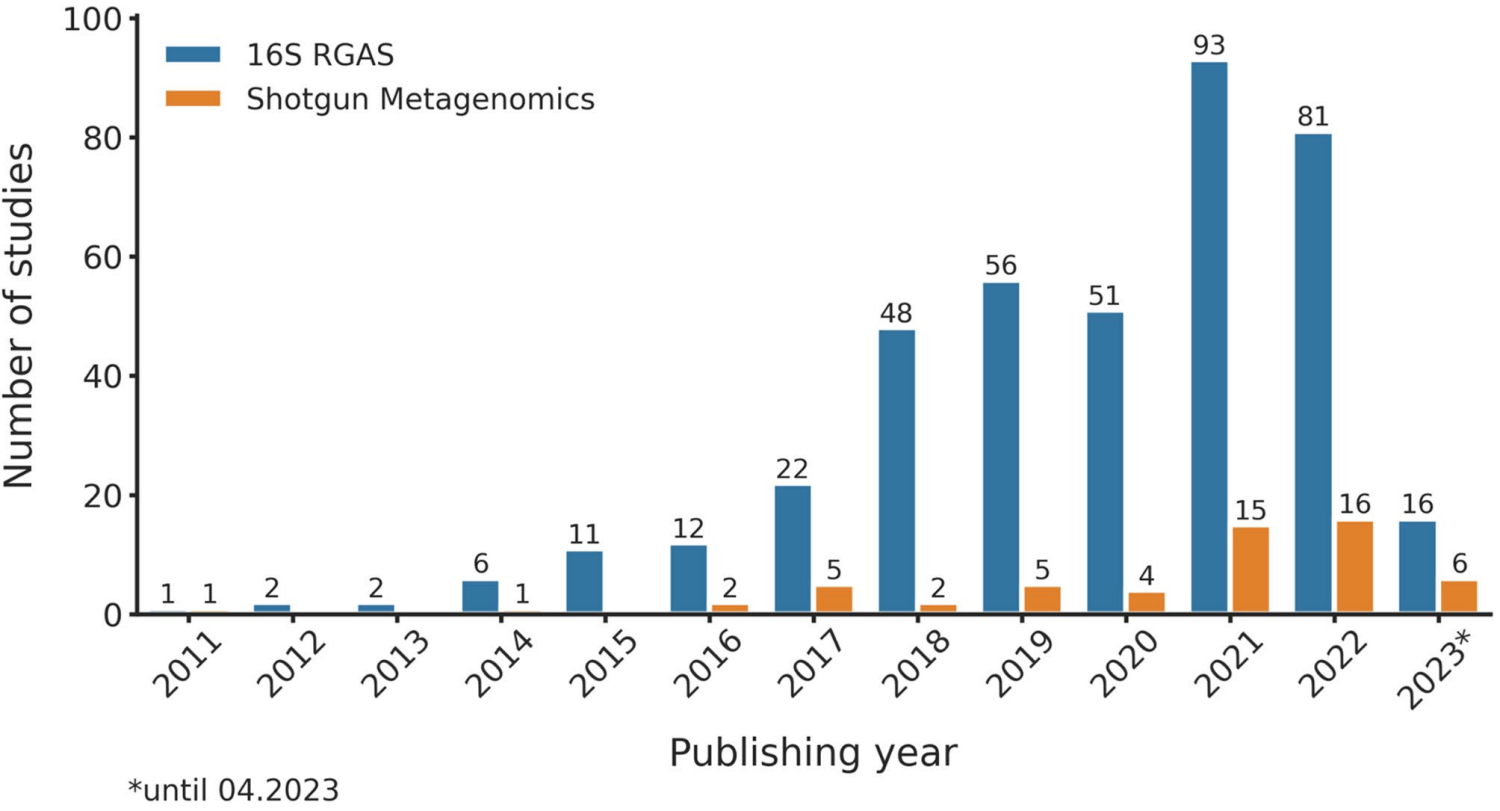
Yearly trend of 16s RGAS based & shotgun metagenomics studies

In recent years, there has been a noticeable increase in the use of shotgun metagenomics (Fig. 4), driven by decreasing sequencing costs and advances in computational analysis [82]. Unlike targeted amplicon sequencing, shotgun metagenomics involves sequencing the entire genomic content of all microorganisms present in a sample [83]. This approach offers deeper insights, enabling species-level taxonomic resolution and the study of non-bacterial community members such as fungi, viruses, and eukaryotes [79]. Additionally, shotgun metagenomics provides valuable information on the functional potential of the microbiome. However, these benefits come at the expense of higher sequencing costs, increased computational demands, and a greater reliance on comprehensive and up-to-date reference databases [84].

Both approaches have distinct methodological considerations. 16S RGAS relies on PCR amplification of specific variable regions using degenerate primers designed to capture a broad range of taxa [85]. The resulting amplicons are sequenced, and taxonomic assignments are made by comparing sequence variation in less-conserved regions to curated databases [86, 87]. While this technique is reliable and well-established, it is limited by the fact that only a single region of the bacterial genome is analyzed, which can result in lower taxonomic resolution and primer-specific biases [3, 88]. In contrast, shotgun metagenomics generates a much larger dataset, often 10 to 30 million reads per sample [85], which supports more precise taxonomic and functional profiling but requires more sophisticated data analysis pipelines [89].

**Fig. 5:**
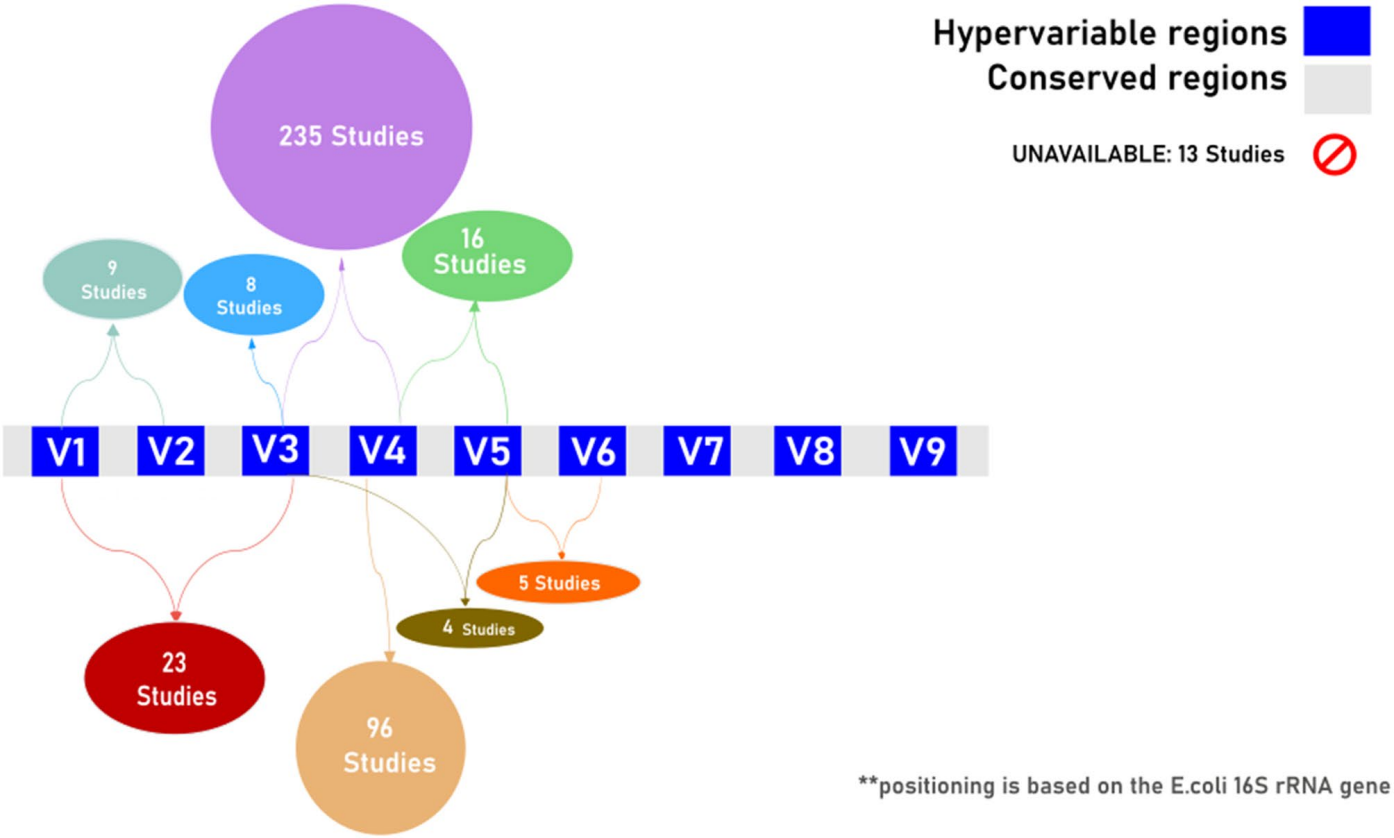
Number of studies using each amplified 16 S rRNA gene region

A key aspect of 16S RGAS is the selection of the variable region targeted for amplification. The 16S rRNA gene is approximately 1500 bp in length and contains nine variable regions interspersed with conserved regions, each with different discriminatory power for various bacterial taxa. For example, the V1–V2 region is less effective at identifying Alphaproteobacteria, while the V3–V5 region underperforms for Actinomycetota (previously Actinobacteria). Conversely, the V6–V9 and V3–V5 regions are better at classifying genera such as Clostridium, Staphylococcus, and Escherichia/Shigella [32]. Our analysis revealed that the V3–V4 region was the most targeted in pig microbiome studies, appearing in 231 publications, followed by the V4 region (106 studies) and the V1–V3 region (23 studies) (Fig. 5). Fourteen studies did not specify which region was amplified. The preference for the V3–V4 region likely reflects its recommendation for human studies due to their ability to capture a broad spectrum of microbial diversity [90].

Despite its strengths, 16S RGAS is limited in its ability to profile non-bacterial members of the microbiome, such as archaea and eukaryotes, together in a single run, and does not directly provide information on microbial functional capacity, although computational tools are available to predict function from marker gene data [91, 92]. Furthermore, the choice of variable region and primer set can lead to over- or under-representation of specific taxa, and some regions, such as V4–V5, may lack sufficient variability to resolve certain groups [79].

### Sequencing platforms

DNA sequencing platforms have evolved rapidly over the past two decades [93]. The introduction of next-generation sequencing (NGS) technologies, such as Illumina, Oxford Nanopore, and Pacific Biosciences, has enabled researchers to sequence millions to billions of DNA fragments in parallel, revolutionizing both the scale and resolution of genomic studies [94]. Early advancements began with Sanger sequencing and the commercial launch of the ABI 370 in 1987, which automated chain-termination sequencing using fluorescently labeled dideoxynucleotides and capillary electrophoresis [95–97]. The second generation of DNA sequencing platforms further transformed the field by allowing for the simultaneous sequencing of thousands to millions of DNA fragments [93]. Some examples include the Roche 454, which utilized pyrosequencing to detect nucleotide incorporation, and the Ion Torrent, which measures hydrogen ion release during DNA synthesis. The widely adopted Illumina platform uses a sequencing-by-synthesis approach with reversible dye terminators, while the SOLiD system employs oligonucleotide ligation and detection. These technologies dramatically increased sequencing speed and throughput, making large-scale and high-resolution studies feasible [93]. The most recent, third-generation platforms, such as PacBio and Oxford Nanopore, have introduced innovative strategies to overcome the limitations of earlier systems, particularly by enabling the sequencing of much longer DNA fragments [93]. PacBio’s single-molecule real-time (SMRT) sequencing and Oxford Nanopore’s nanopore-based technology both allow for real-time analysis and extended read lengths, with the latter detecting changes in electrical current as DNA passes through a nanopore [98].

Our data show that the Illumina MiSeq platform is the most used sequencing platform in pig microbiome studies (*n* = 284). The Illumina HiSeq (85 studies), NovaSeq (39 studies), and Roche 454 GS-FLX+ (28 studies) platforms were the next most frequently used. At the same time, the Illumina NextSeq and Ion Torrent sequencers were used less often and tied for a distant fifth place. Only one study did not report on the sequencing platform used.

Performance comparisons between sequencing technologies and bioinformatics pipelines have shown that high-quality, comparable data can be generated by the Roche GS FLX+, Illumina MiSeq, and Ion Torrent PGM platforms [99–102]. For example, a direct comparison of the Illumina Genome Analyzer (GA) II and Roche GS FLX platforms using identical DNA samples from a freshwater planktonic community found similar overall diversity. However, the Roche GS FLX+ platform delivered more homopolymer errors, while the Illumina GA II produced longer and more accurate contigs [103]. Similarly, studies comparing the V3–V4 16S rRNA gene hypervariable region in the healthy skin microbiome using both Illumina MiSeq and Roche GS FLX+ platforms found concordant results between replicates and across platforms, indicating that both methods are suitable for microbiota profiling [104]. Further comparisons among Roche GS FLX+, Illumina MiSeq, Illumina HiSeq, and Ion Torrent PGM revealed that Ion Torrent produced the longest reads (up to 600 bp), but had a higher error rate in homopolymeric regions [105]. Illumina platforms, in contrast, offered faster run times, higher throughput, and lower per-base costs, though with shorter read lengths and a slightly higher substitution error rate compared to Roche GS FLX+ [106]. Additional studies have reported that Ion Torrent’s homopolymer error rates are lower than those of Roche GS FLX+, but Ion Torrent generates shorter reads, lower throughput, and lower quality scores overall [107]. Analysis of the cecal microbiota using the 16S RGAS on the Illumina MiSeq, Ion Torrent PGM and Roche 454 GS FLX Titanium sequencers found that MiSeq produced the highest amount of reads after quality filtering, GS FLX+ generated the longest reads and highest quality scores, and the Ion Torrent PGM generated data maintained stable quality scores throughout the read length. Overall, the study concluded that these sequencing platforms produced comparable microbiome compositional profiles [108]. Although Roche discontinued support for the 454 GS FLX+ platform in 2016, data generated by this technology remains valuable for retrospective studies. The choice of sequencing platform can influence downstream analyses. For example, Illumina HiSeq is well-suited for deep sequencing, while Illumina MiSeq and Ion Torrent PGM are preferred for functional gene characterization due to their longer read lengths [109]. Given these technical differences, we should avoid direct comparison of data generated from different sequencing platforms unless appropriate bioinformatic tools are used to reconcile platform-specific biases. When selecting a sequencing platform, several criteria must be considered, including sequencing cost, read length, error rates, and throughput. Short-read platforms such as Illumina are currently the most widely adopted due to their affordability and high throughput, making them ideal for detecting single-nucleotide variants and small insertions or deletions. In contrast, long-read platforms such as PacBio and Oxford Nanopore offer longer reads, are top at resolving complex genomic regions and structural variants, but incur higher error rates and costs [110].

### Bioinformatic Pipelines

The choice of bioinformatic pipelines is determinant in the quality, reproducibility, and interpretability of microbiome data. Our review found that the most widely used platforms for analyzing 16S RGAS data in pig microbiome studies are QIIME (including both QIIME 1 and QIIME 2), Mothur, and USEARCH (Table 2). Each of these platforms offers a suite of tools for quality control, clustering, taxonomic assignment, and diversity analyses, but they differ in their underlying algorithms, computational requirements, and default settings. QIIME (QIIME 1 and QIIME 2) and Mothur, the top two most used platforms, have been shown to produce beta-diversity results that cluster samples differently, even when analyzing the same dataset. However, studies indicate that the final number of taxa detected and their relative abundances are generally comparable between the two platforms, with differences often becoming statistically insignificant when up-to-date reference databases are used for taxonomic annotation [111, 112]. This highlights the importance of database selection and versioning, which should always be included in the metadata information.

In our analysis, QIIME pipelines were most frequently paired with the Greengenes database (52 studies) and the SILVA database (46 studies), while Mothur was most often used with SILVA (30 studies) and, to a lesser extent, the now-outdated RDP database (9 studies). USEARCH, the third most used platform, was less likely to have the accompanying database specified, with only four studies reporting the use of SILVA for taxonomic annotation. The major methodological differences between QIIME and Mothur lie in their clustering and classification algorithms. Mothur employs a naïve Bayesian classifier with pseudo-bootstrapping to assign taxonomy, requiring a confidence score above 80%, while QIIME’s approach is based on finding the closest match in the chosen reference database [111, 113, 114]. However, QIIME2’s naive-Bayes, BLAST+ based, and VSEARCH based classifiers have been shown to surpass the taxonomic annotation accuracy of its predecessor [115].

USEARCH, developed by Robert C. Edgar and the third most used platform in pig studies, is valued for its computational efficiency and rapid sequence clustering, taxonomic assignment, and similarity searches [116, 117]. This pipeline features a fast heuristic algorithm that allows for the quick identification of one or a small number of strong hits rather than all homologous sequences to achieve high-throughput [114]. In terms of computational resources, Mothur and USEARCH are less resource-intensive than other tools or pipelines [114, 118]. QIIME2, when using DADA2 or Deblur, requires substantial memory and processing power, especially for large datasets, and has been reported to take several hours to denoise a dataset of over 11 million sequences, even on a high-performance workstation [119].

Despite the widespread use of these pipelines, a significant number of studies in our review did not provide detailed information about their bioinformatic workflows. Specifically, 31 studies did not report the tools used for quality control or denoising, 50 omitted the taxonomic annotation tool, and 53 failed to mention the reference database. Particularly, 45 studies provided no information on either the tool or database used for taxonomic annotation. This lack of transparency impedes reproducibility and limits the ability to compare results across studies.

**Table 2:** Most used platforms for 16 S RGAS analysis

Platform– Tool	Quality Control	Clustering	Taxonomic Annotation Tool	Taxonomic Database
**QIIME (1&2)**	**193 Studies** > DADA2: 31 > Deblur: 6	**120 Studies** > DADA2 (QIIME2) : 33 > Deblur(QIIME2) : 6 > UCLUST (QIIME 1): 8 > USEARCH (QIIME 1): 4	**109 Studies** > QIIME 2: 50 > QIIME 1: 49 > PyNAST (QIIME 1): 2 > RDP Classifier: 1 **Wrongly Classified:** > DADA2 (QIIME2) – 2 > Deblur (QIIME2) – 1 > UCLUST (QIIME 1) – 2	**> Greengenes: 52** ** > SILVA: 46**
**Mothur**	**52 Studies** > Mothur MiSeq SOP: 15 > AmpliconNoise: 3 > UCHIME: 2 VSEARCH: 1	**49 Studies** > Mothur MiSeq SOP: 15 > Opticlust: 1	**36 Studies** > Mothur MiSeq SOP: 7 > RDP Classifier: 1	**> SILVA: 30** ** > RDP: 9**
**USEARCH**	**27 Studies** > UCHIME: 4	**34 Studies** > UPARSE: 1	**6 Studies**	**> SILVA: 4**
**Unavailable**	**31 Studies**	**25 Studies**	**48 Studies**	**50 Studies**
			**No Taxonomy Annotation tool & Database: 45 Studies**	

#### Quality control of sequenced reads

Downstream sequence analysis can be compromised by several factors, such as sequencing artifacts, low-quality reads, and contamination, and, in the case of 16S RGAS, insufficient length of paired reads for merging based on the overlapping region. Such issues may eventually lead to erroneous sequence assembly and inaccurate taxonomic classification, or biases towards microbes with shorter targeted 16S rRNA gene regions. To mitigate these risks, the proper read preprocessing should be applied. For metagenomic studies, quality control (QC) and host/feed DNA removal steps are essential; they enable the assessment of sequence length and quality, GC content, sequence duplication rates, complexity distributions, and the presence of ambiguous bases [120]. For 16S RGAS analyses, especially coupled with Dada2 denoising, removing biological primers is needed and should be preferred over trimming a fixed number of base pairs to exclude artificial inflation of diversity metrics.

Our review of QC tools used in 16S RGAS shows the widespread adoption of QIIME and its successor, QIIME2. Since January 2018, QIIME is no longer supported, with QIIME2 now offering enhanced quality control, resulting in a higher number of high-quality reads after trimming [121].

DADA2, integrated within QIIME2 and also available as an R package, was another tool used in 65 studies for quality control, denoising, and chimeras’ removal. DADA2 is recognized for its ability to model and correct sequencing errors more accurately, reducing the risk of artificially inflating bacterial richness in the analyzed samples [122]. Denoising, the recently developed methodology used by DADA2 is designed to characterize true sequences in a collection of samples based solely on the algorithm’s choices and not on the 97% similarity threshold typically used by OTU focused studies [123].

UCHIME was the second most used tool for chimeras’ detection and removal among 16S RGA sequences. It was shown to be effective, particularly in noisy, short sequences and multimeric amplicons, outperforming earlier tools such as ChimeraSlayer [124]. UCHIME can operate in de novo mode, requiring estimates of unique amplicon sequences and their abundances, or in reference mode, which depends on a comprehensive and phylogenetically diverse database. Constructing such reliable references and accurately estimating sequence abundances remain challenges for users [124].

Mothur, used in 46 studies for quality control, is a robust tool for microbial ecology analysis and phylogenetics [125]; amongst these studies, 14 studies clearly indicated utilizing the Mothur MiSeq SOP [126] to perform quality control. AmpliconNoise [127], UCHIME and VSEARCH were used by individual studies, to carry out this step, and the remaining 29 studies utilizing this platform did not specify what tool or protocol was used to carry out quality control. While Mothur shares many functionalities with QIIME, comparative studies have found that it tends to yield more unassigned reads than QIIME or Bioconductor when using the SILVA database for read classification [29, 125]. It is important to highlight that 31 studies did not report which quality control or chimeras’ detection tools were used.

For quality control and preprocessing of shotgun metagenomic sequenced reads, Trimmomatic (14 studies) was the most used tool, as it is a versatile, pair-aware preprocessing tool optimized for Illumina NGS data [128]. Fastp (8 studies), ranking second in the category, is a rapid preprocessing and QC tool for FASTQ files, reported to be two to five times faster than other tools such as Trimmomatic and Cutadapt [129]. To remove host DNA, 25 studies failed to provide any information on what tools they utilized. Among the tools that were counted in this category, Bowtie2 (13 studies) and BWA (10 studies) were the most used by publications. Both tools, popular for their utility in aligning sequenced reads to reference genomes, have been shown to be effective and accurate in contaminant host DNA removal [130]. To assemble shotgun metagenomic data, MEGAHIT, a de novo assembler for NGS [131], was the most commonly used tool for assembly. This tool is a memory-efficient de novo assembler suitable for both gene- and genome-focused studies, and is particularly recommended for conservative assembly strategies when computational resources are limited [132]. SOAPdenovo and its successor SOAPdenovo2 were also frequently used for de novo genome assembly, with SOAPdenovo2 offering reduced memory usage, increased coverage, and longer scaffolds, making it suitable for larger genomes [133].

#### Clustering and gene prediction tools for 16 S RGA and shotgun metagenomic sequencing data

Over recent years, there has been a great shift from traditional sequence identity-based clustering methods, which generate operational taxonomic units (OTUs), to denoising algorithms that produce amplicon sequence variants (ASVs) [134]. OTUs are typically defined as groups of sequences sharing greater than 97% identity [135], a strategy that reduces dataset size and computational demands while also minimizing the impact of sequencing errors by clustering erroneous reads with their correct counterparts [136, 137]. However, this approach can mask subtle biological differences and may merge distinct taxa into a single OTU. In contrast, ASV-based methods use sophisticated denoising algorithms to distinguish true biological sequences from errors, based on the expectation that true biological reads are repeatedly observed across samples [138]. These methods offer higher resolution and reproducibility, enabling finer-scale ecological and evolutionary analyses. Nevertheless, the impact of denoising pipelines on ecological interpretation, especially in relation to other methodological choices such as rarefaction and diversity index calculation, remains unclear [134].

Our review identified the most widely used tools for clustering/denoising and classifying 16S RGAS and shotgun metagenomic sequences in pig microbiome studies. For 16S RGAS, QIIME and QIIME2 were the predominant platforms. QIIME2, in particular, includes the denoising tools DADA2 and Deblur [139], with DADA2 demonstrating better sensitivity and resolution in mock community tests, albeit with a tendency to generate some false ASVs [29]. UPARSE was the second most used tool for OTU clustering in 16S RGAS studie. UPARSE employs a maximum expected error quality filtering strategy rather than average quality, and has been reported to outperform Mothur, QIIME, and AmpliconNoise in recovering sequence clusters from mock communities [140]. Mothur, the third most used clustering tool, produces results comparable to QIIME2 but has been noted for generating more false positives [141]. USEARCH, often paired with the UCLUST algorithm, was also frequently used for clustering, utilizing efficient algorithms for both global and local sequence alignments [114].

For shotgun metagenomic data, the clustering of microbial genomes after metagenome assembly and binning enables comprehensive analysis of both cultured and uncultured microbes, providing new insights into microbial community structure and metabolic pathways [82, 142]. Among the tools used, CD-HIT stood out as the most popular for de novo assembly of shotgun metagenomic sequences. Originally developed for protein sequence assembly and later adapted for nucleotide sequences [143]. Recent developments in bioinformatics, such as the release of MOSHPIT, a software suite built on the QIIME2 framework for untargeted whole metagenomics, may help standardize analyses in the future [144]. However, as it was released recently, its adoption in pig microbiome research remains limited at this time. CD-HIT is known for its ability to recover a high proportion of true genome fragments, with simulations showing close agreement between assembled and original genome fragments except in cases of high allelic diversity [145]. MetaGeneMark, a gene prediction tool, was also used significantly on assembled reads (15 studies). Its algorithm uses GC content-specific models to predict protein-coding regions in unidentified sequence fragments as short as 400 nucleotides [146]. Metagene is an outmoded version of MetaGeneMark, which was also designed for gene prediction in genomic sequences [142]. Other gene prediction tools, such as Prodigal, were also utilized in four studies. This tool identifies genes and translation initiation sites in microbial genomes, with performance comparable to or better than other leading gene prediction tools such as Glimmer, EasyGene, and GenemarkHMM [147].

#### Taxonomic annotation databases and software

Selecting an appropriate reference database and annotation tool is an important step for accurate taxonomic classification in microbiome studies. Our review highlights that the SILVA (174 studies) and Greengenes (121 studies) databases are by far the most widely used resources for 16S RGAS-based studies in pigs [148], with the RDP database also frequently cited (54 studies). These databases serve as repositories for taxonomic annotation, each offering unique strengths and coverage.

The SILVA database is especially valued for its comprehensive, manually curated taxonomic data covering Bacteria, Archaea, and Eukarya, primarily based on small subunit rRNA gene sequences (16S for prokaryotes and 18 S for eukaryotes) [149]. Since its inception in 1991 as part of the ARB project, SILVA has been regularly updated, evolving into a leading reference for rRNA gene sequences [150, 151]. Each sequence in SILVA is enriched with contextual information, including type strain data, current nomenclature, and taxonomic classifications from LTP, RDP, Greengenes, and INSDC. Quality control is rigorous, and extensive documentation is available for every release, ensuring transparency and reliability [150, 152]. The Greengenes database, dedicated to Bacteria and Archaea ^155^, builds its taxonomy on de novo phylogenetic tree construction, integrating rankings from major taxonomic databases, particularly NCBI [153]. The latest version, Greengenes2 (released in 2024), merges whole-genome and 16S RGA data into a unified reference tree, significantly improving consistency across microbiome studies. With over 21 million sequences from initiatives like the Earth Microbiome Project and the American Gut Project, Greengenes2 enhances compatibility between 16S RGAS and shotgun metagenomic data and is now updated regularly [154].

The RDP database, which includes sequences from Bacteria, Archaea, and Fungi sourced from the International Nucleotide Sequence Database Collaboration (INSDC), is another important resource [155]. Its taxonomic framework is based on Bergey’s Manual^27^ and the List of Prokaryotic Names with Standing in Nomenclature (LPSN) [156] for prokaryotes, and hand-curated for fungi. However, the RDP database is no longer being updated like SILVA or GreenGenes2, and this will limit its accuracy for the most current taxonomic assignments [157]. The RDP Classifier, which utilizes the RDP reference dataset, is the most used tool. This classifier employs a fast, memory-efficient, and alignment-free approach, using bootstrap confidence values to assign taxonomy [158]. While it is well-suited for high-throughput analysis and partial sequences, its reliable classification typically extends only to the genus level due to the conserved nature of rRNA gene sequences [159]. However, given the outmoded nature of the RDP database and the continuous increase in the adoption of the QIIME platform, our review concludes that QIIME and QIIME2 should be categorized as the most used tools for taxonomic annotation of 16S RGAS. The plugin for taxonomy classification in QIIME2 allows for the use of a number of machine-learning classifiers, like those available in scikit-learn, for taxonomic annotation based on marker genes, and also offers two alignment based classifiers built on BLAST and VSEARCH [115].

A comparison of GreenGenes, SILVA, RDP and NCBI evaluating how these databases influence 16S RGAS microbiota profiling results showed that alpha diversity estimates varied significantly across databases with SILVA consistently showing the lowest phylogenetic diversity while RDP and NCBI produced higher values; however, all databases were able to preserve the original experimental group differences in both alpha and beta diversity metrics [160]. At the genus level, the choice of reference database for 16S RGAS studies has also been shown to be a potential factor that can introduce bias [157]. Variations in taxonomic annotations at this level affect relative abundance metrics and the annotation accuracy is typically dependent on which database is the most recently updated [157, 160].

For shotgun metagenomic data, the NCBI databases (NCBI-NR, NCBI-NT and NCBI-RefSeq) are the most frequently used reference databases. The NCBI taxonomy is manually curated using current systematic literature and over 150 sources, but it contains some duplicate names for different organisms [161]. The Genome Taxonomy Database (GTDB) is also widely used, with its associated toolkit (GTDB-tk) ranking as the third most common tool for taxonomic annotation in shotgun metagenomics. GTDB is updated twice annually, ensuring inclusion of the latest genomes from NCBI Assembly [162]. GTDB-tk, although computationally demanding, enables efficient annotation of large genome sets [163]. MG-RAST, which uses its own M5rna database integrating SILVA, GreenGenes, and RDP, is also popular for shotgun metagenomic annotation [164]. DIAMOND [165], a high-speed sequence similarity search tool, is the most used for annotating shotgun metagenomic sequences. It can align large datasets of short or long reads against extensive reference databases like NCBI-nr, provided sufficient computational resources are available [166–168]. CD-HIT and GTDB-tk are also frequently employed in this context. BLAST remains a staple for taxonomic annotation in shotgun metagenomics, using sequence similarity searches against databases classified with NCBI taxonomy [169]. The frequent pairing of the Greengenes and SILVA databases with QIIME as the annotation tool highlights a strong synergy between these databases and QIIME’s versatility for taxonomic assignments. In contrast, tools such as RDP Classifier, Mothur, and DIAMOND have greater specificity, as they are predominantly associated with a single primary database rather than being used interchangeably.

Our analysis also revealed significant gaps in reporting: 64 studies (50 using 16 S RGAS and 14 using shotgun metagenomics) did not specify which database was used for taxonomic annotation, while 55 studies (48 studies using 16 S RGAS and 7 using shotgun metagenomics) failed to report which annotation tool was employed. A total of 37 studies utilizing 16 S RGAS provided no information on either the tool or database used for taxonomic annotation.

#### Functional annotation databases and tools

While 16S RGAS-based studies are highly effective for characterizing the phylogenetic composition of microbial communities, they provide limited information about the metabolic capabilities of those communities. As a result, predicting the functional potential of a microbiome, especially from marker gene data, remains a significant challenge, since the genomes and corresponding functional repertoires of many organisms represented in these databases are still unknown [170]. The accuracy of functional gene predictions is further influenced by the underlying taxonomic composition of the community [171]. Therefore, identifying genes within genomic sequences using shotgun metagenomics is necessary for understanding and annotating the functional potential of prokaryotic communities [171].

Among the studies that utilize shotgun metagenomic sequencing, the Kyoto Encyclopedia of Genes and Genomes (KEGG) stands out as the most frequently used database for pathway and functional annotation in pig microbiome studies (20 studies). KEGG is designed to link genomic information with functional insights by standardizing gene annotations and mapping them to metabolic pathways [172]. The next most used databases are the Carbohydrate-Active enZymes (CAZy) database (12 studies) and the Comprehensive Antibiotic Resistance Database (CARD) (12 studies). CAZy provides continuously updated classifications of enzyme families involved in the synthesis, modification, and degradation of oligo- and polysaccharides. This resource is very effective for studies exploring carbohydrate metabolism within the gut microbiome [173], whilst CARD offers the Antibiotic Resistance Ontology (ARO), enabling rapid detection of potential antibiotic resistance genes in newly sequenced or unannotated genomes [174].

In functional annotation, the most adopted approach is the comparison of predicted query proteins to existing databases of protein sequences [175], such as the aforementioned functional annotation databases. To perform this comparison, BLAST and its ensuing variants (BLASTX, BLASTP, etc.) used by 13 publications in this review, are the most widely adopted algorithms for this purpose [176]. It is important to note that homology-based algorithms like BLAST typically suffer from prolonged computation times needed to identify homologs for each sequence within typically enormous metagenomic datasets. Additionally, the requirement for the searched database to contain at least one homology of the queried sequence and the discontinous nature of shotgun metagenomic sequences negatively affect the accuracy of the homology-based functional annotation [175]. DIAMOND, the second tool in this category (6 studies), has been touted as possibly the fastest tool in this area based on its usage of double indexing on both the query sequence and the reference database [177]. With its new and unique algorithm, DIAMOND has been shown to match the sensitivity of BLAST’s homolog queries whilst being significantly more efficient [178].

## Recommendations for metadata collection and reporting

A metadata standard, as defined by the International Organization for Standardization, is a complete document that provides a unified approach to organizing and interpreting data, outlining both the principles and practical considerations for implementation [38]. Such standards are intended to ensure data consistency, interoperability, and reproducibility across different studies and platforms. In the context of pig microbiome research, standardized metadata collection is especially important given the wide variability in experimental design, sample handling, sequencing, and analysis protocols observed in the literature. To address these challenges, we propose a metadata template for pig microbiome studies, adapted from the MIMARKS-C checklist under the Minimum Information about any (x) Sequence (MIxS) standards developed by the Genomic Standards Consortium [37], and further refined for the specific needs of livestock microbiome research. This template is designed to facilitate the systematic collection and reporting of metadata, thereby enhancing the comparability and integration of data across studies.

**Fig. 6:**
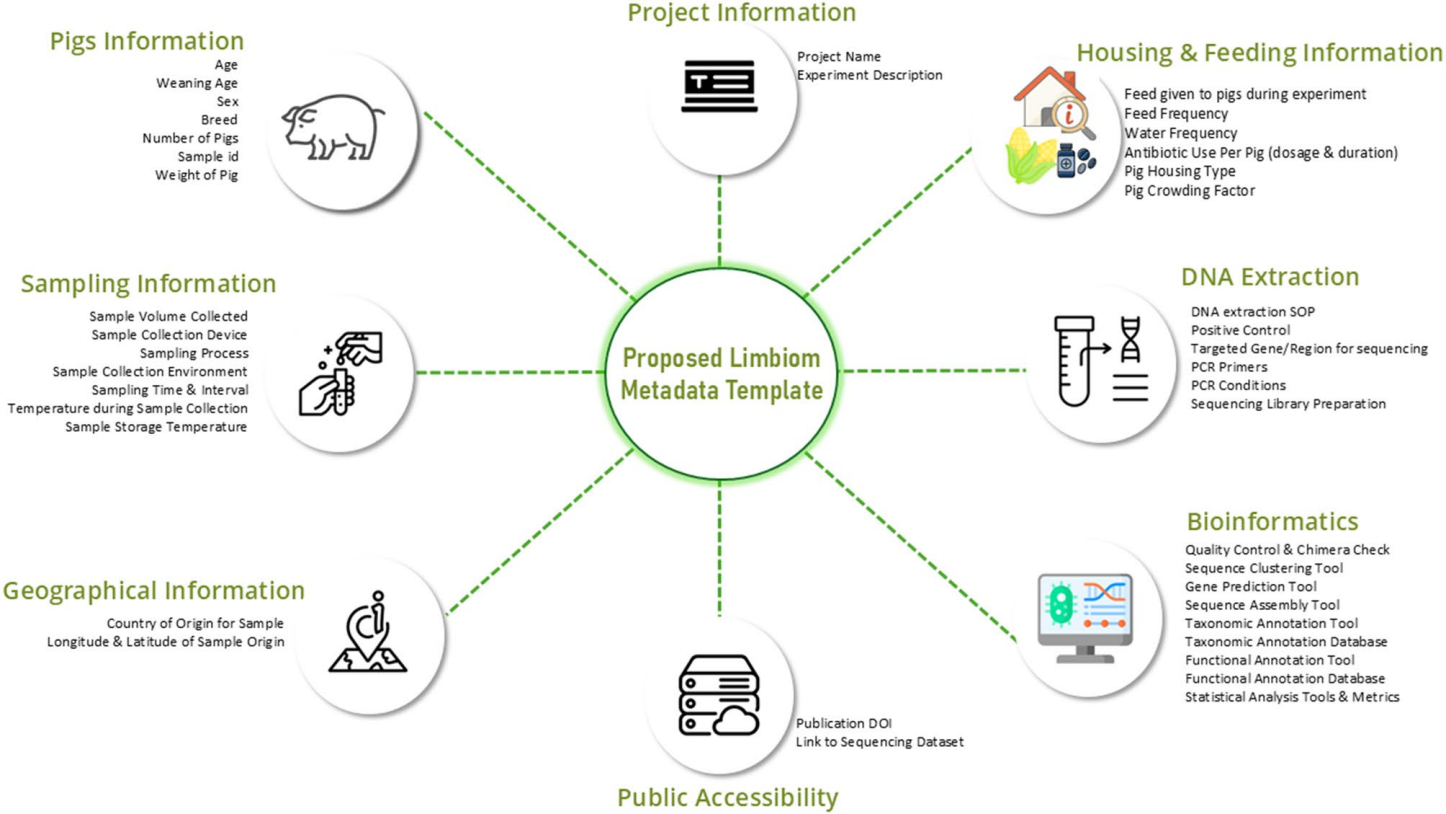
Categories of proposed metadata template

Our proposed template (Supplementary File 2) contains key metadata fields that combines standardized columns recommended by MIMARKS-C with additional fields reflecting all the parameters analyzed in the publications included in this review. These fields capture essential information on sample collection, experimental design, animal characteristics, DNA extraction, sequencing, and bioinformatics workflows (Fig. 6). By integrating both universal and study-derived metadata, this template supports well-organized, reproducible, and interoperable datasets that can be readily shared and reused within the research community.

**Fig. 7:**
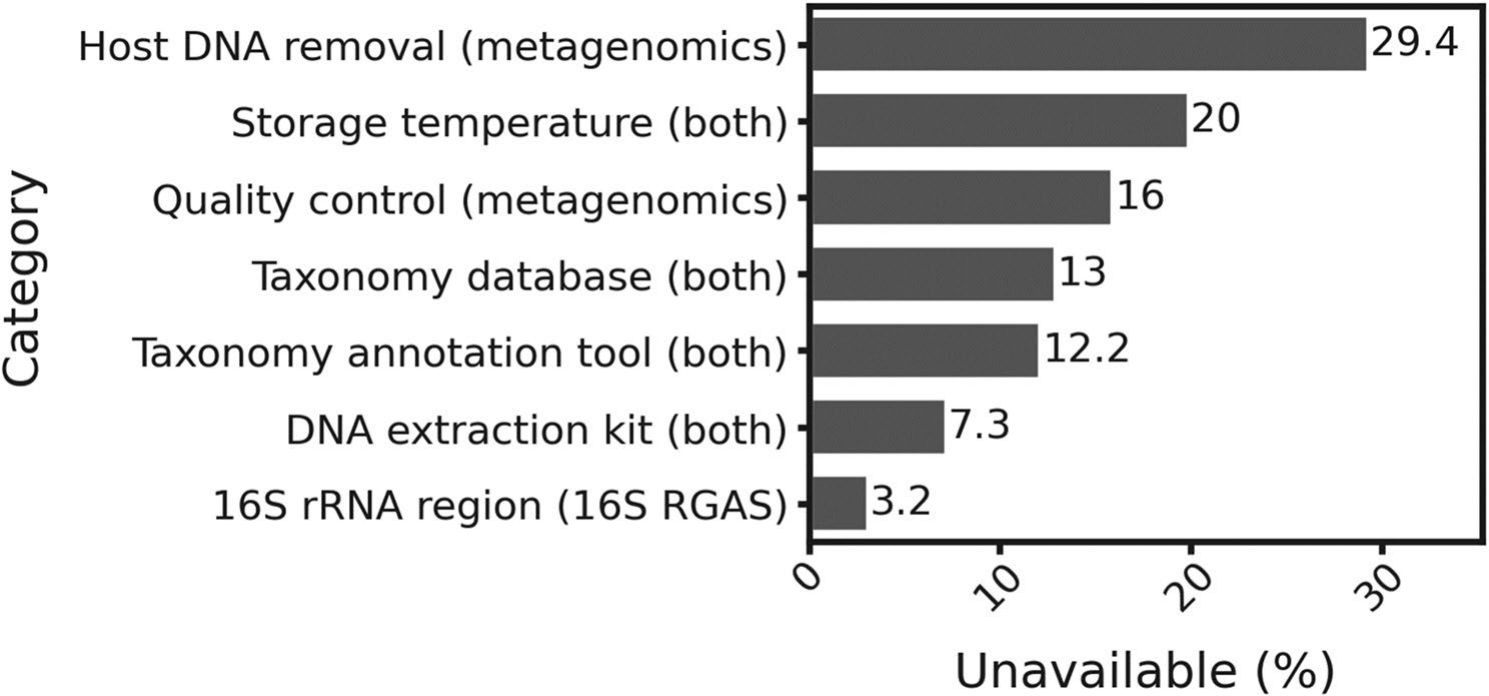
Percentage of the studies with unavailable metadata by category and study type

In our review, we found that many studies failed to report crucial information regarding data processing (Fig. 7), which negatively impacts data interpretation and contradicts the principles of FAIR (Findable, Accessible, Interoperable, Reusable) data. Among the studies that used shotgun metagenomics sequencing, nearly 30% did not report the tool used for host DNA removal, and 16% either omitted the quality control tool or failed to specify whether quality filtering was performed. In studies using the RGAS approach, 3.2% did not specify the targeted hypervariable region of the 16 S rRNA gene. In general, across all studies included in this review, 20% did not report the storage temperature of the samples prior to processing, 13% and 12.2% failed to mention the database and tool used for taxonomy annotation, respectively, and 7.2% did not disclose the DNA extraction kit. Therefore, we recommend adopting this standardized metadata reporting template as a minimum requirement for pig microbiome studies. Consistent implementation of such standards will improve transparency and reproducibility, facilitate meta-analyses and cross-study comparisons, support compliance with FAIR data principles, and enable more robust and meaningful scientific conclusions.

Implementing standardized metadata reporting may require adjustments to current laboratory and data management practices. However, the long-term benefits, including increased data utility, improved research integrity, and enhanced opportunities for collaboration, far outweigh the initial effort. We also encourage journals, funding agencies, and data repositories to require or incentivize the use of standardized metadata templates in pig microbiome research submissions.

## Challenges and future perspectives

Despite significant progress in pig microbiome research, several challenges continue to limit the field’s progress and impact. One of the most pressing issues is the limited availability and accessibility of sequencing data and associated metadata. Many studies fail to deposit raw data or provide insufficient contextual information, severely limiting opportunities for systematic reviews, meta-analyses, and independent validation. This is a widespread issue in microbiome research and highlights the urgent need for adherence to FAIR data principles.

Technical obstacles also persist, including variability in sampling methods, DNA extraction protocols, sequencing platforms, and bioinformatic pipelines. These inconsistencies can introduce bias and limit the comparability of results across studies. The rapid evolution of sequencing technologies and analytical tools further complicates standardization efforts, underscoring the importance of adaptable protocols and ongoing methodological evaluation. Additionally, the varying levels of adoption of MIxS environmental packages such as the MIMARKS-C by repositories such as the Sequence Read Archive (SRA) and the European Nucleotide Archive (ENA) indicate distinct user communities amongst these repositories; in the ENA, MIxS packages are more utilized across studies than samples types indicating that a majority of smaller studies implement MIxS metadata reporting standards while in the SRA, human-associated metadata reporting standards are more widely used [179]. These variations do not imply that the default checklists of these repositories are incorrect or poorly curated. Instead, it highlights the need for greater collaboration between the Genome Standards Consortium and these repositories to harmonize metadata collection checklists across different sample and study types.

Standardizing metadata collection and reporting is another critical challenge. While our proposed metadata template offers a structured approach, its adoption may be hindered by the need to adapt existing laboratory workflows, the diversity of experimental designs, and resource limitations, particularly in smaller research groups. Data privacy concerns, such as protecting proprietary farming practices or sensitive genetic information, add another layer of complexity. Similar challenges have been noted in other areas of microbiome science, emphasizing the need for community-driven solutions and flexible standards that can evolve with the field. Implementing a standardized metadata template, as proposed in this review, represents a step forward for more organized and reproducible data collection and reporting.

Addressing these challenges will require a multifaceted approach. Training and education initiatives are important to raise awareness of the benefits of standardized metadata collection and to provide practical guidance for implementation. Integrating metadata templates into established data repositories, such as the NCBI Sequence Read Archive or EMBL-EBI’s MGnify, could facilitate the submission of complete and consistent metadata alongside sequencing data, further supporting data harmonization and reuse.

In conclusion, although some progress has been made in characterizing the pig microbiome and standardizing research practices, further efforts are necessary to establish unified protocols, enhance data accessibility, improve metadata reporting, and foster collaboration within the research community. Addressing these challenges will support new discoveries and enable more reproducible, impactful, and translational pig microbiome research in the future.

## Supplementary Information

Below is the link to the electronic supplementary material.

Supplementary Material 1

Supplementary Material 2

## Data Availability

No datasets were generated or analysed during the current study.

## Abbreviations

16S RGAS: 16S rRNA Sequencing
DNA: Deoxyribonucleic Acid
PRISMA: Preferred Reporting Items for Systematic reviews and Meta-Analyses
RCTs: Randomized Controlled Trials
PBS: Phosphate-buffered Saline
RNA: Ribonucleic Acid
FTA: Fast Technology for Analysis of Nucleic Acids
FOBT: Fecal Occult Blood Tests
CTAB: Conventional Hexadecyltrimethylammonium Bromide
PCR: Polymerase Chain Reactions
EDTA: Ethylenediaminetetraacetic acid
NGS: Next-generation Sequencing
SMRT: Single-Molecule Real-Time
QC: Quality Control
OTUs: Operational Taxonomic Units
ASVs: Amplicon Sequence Variants
INSDC: International Nucleotide Sequence Database Collaboration
NCBI: National Center for Biotechnology Information
BLAST: Basic Local Alignment Search Tool
KEGG: Kyoto Encyclopedia of Genes and Genomes
CAZy: Carbohydrate-Active enZymes
CARD: Comprehensive Antibiotic Resistance Database
MIxS: Minimum Information about any (x) Sequence standard
MIMARCKS: C-Minimal Information about a Marker Sequence: specimen
FAIR: Findable, Accessible, Interoperable, Reusable
